# Can a naturally depauperate Ephemeroptera, Plectoptera and Trichoptera (EPT) fauna track river degradation in south-western Australia?

**DOI:** 10.1007/s10661-024-12734-8

**Published:** 2024-06-03

**Authors:** Kathryn R. Greenop, Barbara A. Stewart, Paul G. Close

**Affiliations:** 1 School of Biological Sciences, University of Western Australia, Perth, WA 6009 Australia; 2 Centre for Natural Resource Management, University of Western Australia, Albany, WA 6330 Australia; 3 School of Agricultural and Environmental Science, University of Western Australia, Albany, WA 6330 Australia

**Keywords:** Macroinvertebrates, River health, Index, Biomonitoring, Rapid assessment

## Abstract

Freshwater aquatic ecosystems are threatened globally. Biological monitoring is required to deliver rapid and replicable assessment of changes in habitat quality. The Ephemeroptera, Plectoptera, Trichoptera (EPT) index is a globally recognised rapid bioassessment that measures taxa richness of three insect orders whose larvae are considered sensitive to freshwater habitat degradation. South-western Australia contains threatened freshwater ecosystems but has depauperate EPT fauna and high endemism, potentially reducing the capacity of the EPT index to track degradation. This study investigated if EPT species richness, composition or individual species tracked physical or chemical river degradation in three catchments in south-western Australia. We sampled EPT fauna and measured water chemistry, erosion, sedimentation, riparian vegetation cover and instream habitat at 98 sites in the winters of 2007 and 2023. We found 35 EPT taxa across the study area with a median number of species per site of two. EPT species richness had weak positive associations with a composite water quality index and dissolved oxygen and weak negative associations with electrical conductivity and total nitrogen. No association was found between physical and fringing zone degradation measures and EPT species richness. EPT community structure generally did not distinguish between sites with high or low degradation levels. The presence of the mayfly *Nyungara bunni* tracked salinity, dissolved oxygen and nitrogen levels, but its usefulness as a bioindicator could be limited by its restricted range. This study suggests that the EPT index would need modification or combination with other indices to be a useful rapid bioassessment in south-western Australia.

**Supplementary Information:**

The online version contains supplementary material available at 10.1007/s10661-024-12734-8.

## Introduction

Freshwater aquatic ecosystems are some of the most threatened habitats on the planet (Dudgeon et al., 2006; Olson & Dinerstein, 2002; Vorosmarty et al., 2010). This includes south-western Australian rivers and streams, which are listed as one of the Global 200 Priority Ecoregions and considered endangered (Olson & Dinerstein, 2002) and in ‘Very Poor’ condition (Murphy & van Leeuwen, 2021). Climate change is a particular threat to south-western aquatic habitats due to declining rainfall (Silberstein et al., 2012), which when coupled with the Mediterranean climate and flat terrain results in low runoff and increasingly intermittent stream flow (Bunn et al., 1986; Green & Moggridge, 2021; Murphy & van Leeuwen, 2021). Other serious threats to these habitats include widespread clearing of vegetation, secondary salinity, nutrient runoff and invasive weeds (Halse et al., 2007; Murphy & van Leeuwen, 2021; Stewart, 2011a).

Monitoring these aquatic ecosystems is essential so that ongoing degradation can be identified, and restoration efforts tracked in a meaningful and replicable manner (Dudgeon et al., 2006; South Coast NRM, 2018). Monitoring with physical surveys or chemical analysis alone may not adequately assess biodiversity loss or chronic biotic consequences of pollution or degradation (Karr, 1994; Loeb, 1994). Biological monitoring addresses these issues by measuring changes in species richness and assemblage that may point to a longer-term deterioration in habitat suitability for freshwater organisms (Karr, 1994; Loeb, 1994). The assumptions of this approach are that species richness is sensitive to changes in river conditions and will decline with greater levels of degradation (Karr, 1994).

Bioassessment methods vary; some require non-degraded reference sites and a broad range of assessments, including the identification of all available species. The Australian River Assessment Scheme (AUSRIVAS) uses this methodology to produce a grade of river condition relative to that expected for a ‘pristine’ river with similar physical and biogeographical conditions (van Looij, 2009). However, such assessments are very time-consuming and require considerable taxonomic expertise, making them impractical or inefficient for timely monitoring of large areas (Growns et al., 1997; Lenat & Barbour, 1994). To improve efficiency and reduce lags in reporting time, rapid bioassessments were developed that require smaller subsets of biological information (Growns et al., 1997; Hewlett, 2000; Karr, 1994; Lenat & Barbour, 1994).

One such globally recognised and well-established rapid bioassessment is the EPT (Ephemeroptera, Plecoptera and Trichoptera) index. This index is the taxa richness of these three insect orders (mayflies, stoneflies and caddisflies) whose aquatic larvae are considered sensitive to river degradation (Plafkin et al., 1989). The index was developed in the United States (Plafkin et al., 1989) where it has shown effective discrimination of degraded from non-degraded sites (Lenat & Barbour, 1994; Lydy et al., 2000; Reif, 2002), and it has been effective in Europe (Kaelin & Altermatt, 2016), eastern Australia (Chessman et al., 2006; Growns et al., 1997; Metzeling et al., 2006; Wright & Burgin, 2009), Asia (Wong et al., 2020) and Africa (Akamagwuna et al., 2019). The benefits of using EPT fauna as bioindicators are that the taxa are well-described in taxonomic literature, are visually distinctive in the field and when used as a biotic index generally perform well in sensitivity and efficiency compared to other biological assessments (Growns et al., 1997; Kefford et al., 2022; Metzeling et al., 2006; Wright & Burgin, 2009). Additionally, examining only EPT in aquatic fauna samples limits search and identification effort in the field and laboratory, can be undertaken by non-specialists and community groups and provides simple and replicable reporting metrics (Cox et al., 2019; Hewlett, 2000).

The EPT index could make a useful addition to the monitoring toolbox in south-western Australia given the numerous threats to aquatic habitats in the region, the size of the area to monitor, and the limited resources and expertise needed to interpret the index. However, south-western Australia has a naturally depauperate EPT fauna compared to the Northern Hemisphere and eastern Australia (Bunn & Davies, 1990; Cartwright et al., 2023; Sutcliffe, 2003). For example, Bunn and Davies (1990) found an average of 33 EPT species in 12 families in south-western streams compared to 120 species and 28 families in streams in south-eastern Australia. In eastern Australia, EPT fauna is rich enough that it is usually reported only at the family level but is still effective at tracking river degradation (Hewlett, 2000; Marchant et al., 1995). A depauperate fauna may result in an insufficient range in taxa richness to follow gradients in river conditions, as well as increasing sampling error (Lomond, 1997; Metzeling et al., 2006).

Few studies have directly investigated the utility of the EPT index in naturally depauperate regions. A Canadian study investigated if the EPT index tracked water quality in Newfoundland, where EPT fauna has only 20–40% of the species richness of mainland boreal North America (Lomond, 1997). They concluded that EPT species richness and abundance were effective at distinguishing sites by degradation level. However, Newfoundland has up to 165 EPT taxa (Lomond, 1997), substantially higher than the estimated total across south-western Australia of 67 (Cartwright et al., 2023; Dean, 1999a, 2000; Hynes & Bunn, 1984; Sutcliffe, 2003; Suter, 1999; Webb & Suter, 2011).

Another potential issue with using the EPT index in south-western Australia is that around 70% of the EPT fauna in the region are endemic (Cartwright et al., 2023; Dean, 1999a, 2000; Hynes & Bunn, 1984; Sutcliffe, 2003), and the sensitivity of south-western endemic species to environmental gradients is not well established at a local level. Catchment-scale studies in south-western Australia found reduced EPT species counts in catchments with higher nutrient levels (Stewart, 2011a) and agricultural land compared to natural vegetation (Stewart, 2011b). While not specifically reporting EPT species richness as an outcome, some studies have found mayflies to be more sensitive to low oxygen and salinity relative to stoneflies (Storey et al., 1991). Stoneflies were reportedly absent from the Eastern South Coast Bioregion where river salinity was high (Stewart, 2009) and were poorly represented in a high-salinity river on the west coast of Australia (Bunn & Davies, 1992). These findings suggest that EPT species richness tracks river degradation. However, the endemic stonefly *Newmanoperla exigua* showed tolerance to dam-induced low flow and low oxygen conditions in the Perth hills (Storey et al., 1991). Additionally, caddisfly families in the south-western Australia may vary considerably in their sensitivity, with some studies reporting Hydropsychidae being more tolerant of high salinity or sediment and low oxygen than Hydroptilidae or Leptoceridae (Bunn & Davies, 1992; Storey et al., 1990, 1991). Leptocerid caddisflies and Baetid and Caenid mayflies have also shown unusually high tolerances to salinity in south-western Australia (Kay et al., 2001). These latter studies make it unclear that EPT species richness would reliably decline with the common measures of river degradation. As yet, no study in south-western Australia has directly tested if the EPT index, used in isolation, tracks aquatic degradation gradients.

This study aimed to establish if the EPT index tracks degradation measures despite the limitations of depauperate species richness and high endemicity in a case study on the South Coast Region of south-western Australia. EPT community composition and individual species’ responses were also investigated as these may also change in response to degradation level, and these measures may be particularly useful if absolute EPT species richness is low. The specific hypotheses tested were that (1) EPT index (species count) will decrease with increasing river degradation (as measured with water chemistry and physical assessment), (2) EPT species composition will differ between sites that have high and low levels of chemical and physical degradation and (3) the presence of some individual species will reduce as degradation levels increase.

## Materials and methods

### Study area

**Fig. 1:**
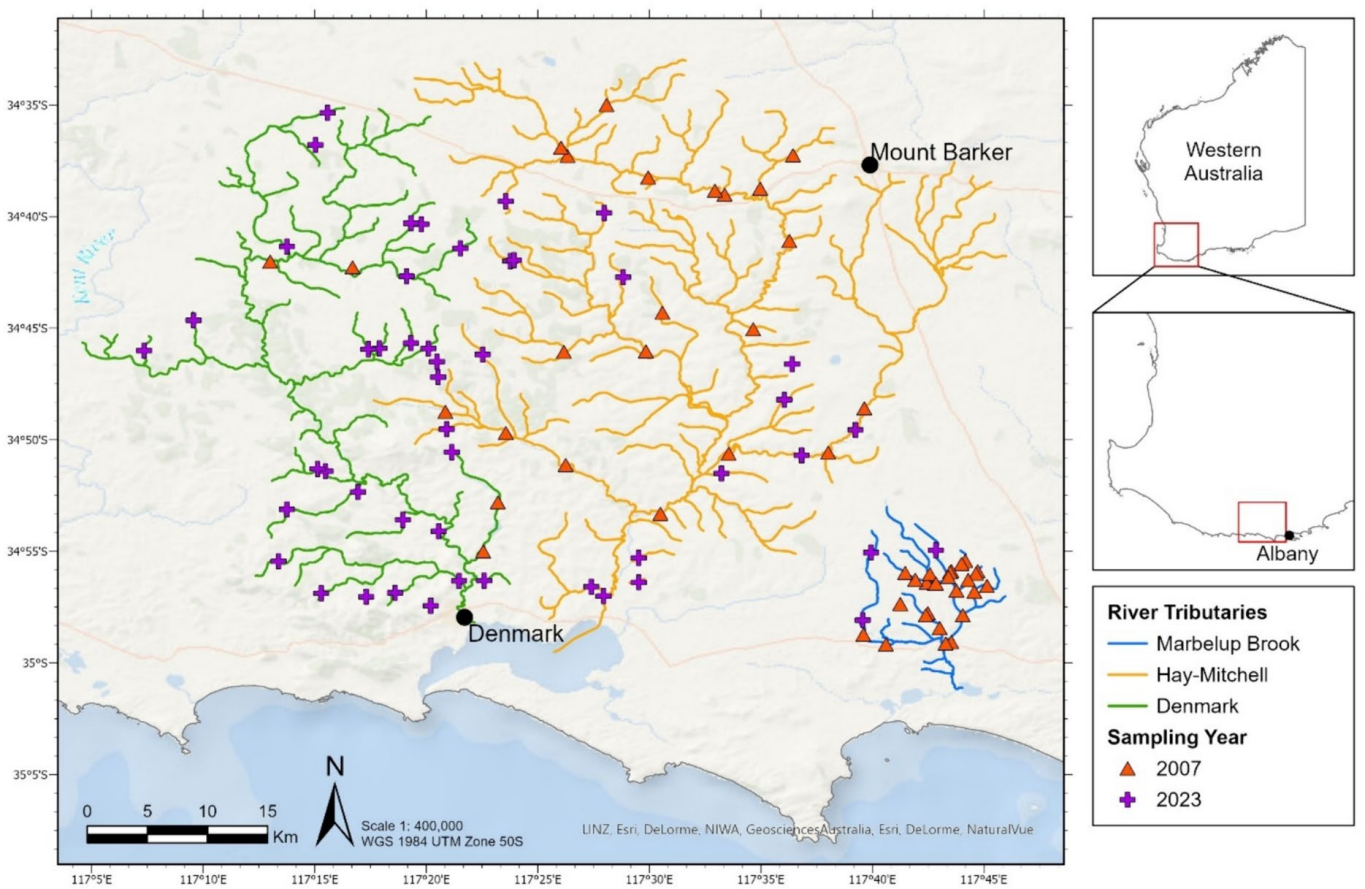
The location of sites sampled in 2007 and 2023 (*N* = 98) for the three study catchments located on the South Coast of Western Australia

We used a case study approach covering three adjacent catchments (Denmark, Hay-Mitchell and Marbelup Brook), spanning an area of 1700 km^2^ on the South Coast Region of Western Australia (Fig. 1). These catchments were chosen to represent similar climactic and biogeographical zones and relatively rich EPT fauna, with the whole study area lying within the Western South Coast Aquatic Bioregion (Stewart, 2009, 2011a). Annual rainfall in the study area ranges from 700 to 900 mm (Department of Water, 2007; Department of Water and Environmental Regulation & Department of Primary Industries and Regional Development, 2021a). Land uses in the study area mainly comprise livestock production, plantation timber and conservation estate (Stewart, 2011a). Approximately 42% of the study area has been cleared of native vegetation although this varies by catchment (22% of Denmark, 55% of Hay-Mitchell and 70% of Marbelup Brook (Department of Water, 2007; Department of Water and Environmental Regulation & Department of Primary Industries and Regional Development, 2021a, b)).

### Site selection

Fifty sites (four in Denmark, 20 in Hay-Mitchell and 26 in Marbelup Brook catchment) previously sampled in the winter of 2007 had suitable data and were included in analyses (aggregated data reported by Stewart, 2011a, 2011b). To reach a sampling size of around 30 sites per catchment, new sites were chosen by assessing maps of watercourses (Crossman & Li, 2015) where previous data was sparse and road access available, while prioritising first and second-order streams to increase independence of observations. Only flowing, shallow sites (< 1.5 m depth) were included. Forty-eight new sites were visited between 29 May and 12 July 2023 (28 in Denmark, 17 in Hay-Mitchell and three in Marbelup Brook catchments) and assessed using the same protocols used during the 2007 surveys, resulting in 98 sites available for analysis (Fig. 1). At each field site, a 10-m sampling reach was identified that avoided pooling near road culverts and the location was plotted by Easting and Northing (UTM Zone 50H, WGS 84) using a ETrex 22 × GPS (Garmin, USA).

### Field data collection

We collected water samples from within each 10-m sampling reach in a triple-rinsed falcon tube that was kept chilled in the field until frozen for transport to the analysis laboratory. Water quality variables (pH, electrical conductivity (EC) in μScm^−1^, salinity in practical salinity units (PSU), turbidity in formazin nephelometric units (FNU)/nephelometric turbidity units (NTU), temperature (in °C), dissolved oxygen (DO) in parts per million (ppm)) were measured with a Hanna Industries multiparameter water quality analyser (model HI 9829 with HI 7609829 probe for 2023 sites and a Yeo-Kal 611 multiparameter water quality analyser (Yeo-Kal, Brookvale, Australia) for 2007 sites. While the 2023 multiparameter used FNU rather than NTU to measure turbidity, for the precision required in this study, these measures were considered equivalent. Total nitrogen (TN) and phosphorous (TP) for 2023 samples (in μgL^−1^) were determined at the University of Western Australia’s Earth and Environment Laboratory using a Lachat QuikChem QC8500 Series 2 FIA Automated Ion Analyser, while samples from 2007 were determined by the Marine and Freshwater Laboratory at Murdoch University, Western Australia using a Lachat Automated Flow Injection Analyser (both Hach, CO, USA) (Stewart, 2011a, 2011b).

A habitat survey was conducted for the 10-m reach and 20 m upstream of this area to visually assess the degree of bank erosion, bed sedimentation, width of fringing vegetation (in metres from bank), proportion of tree/shrub cover and proportion of exotic vegetation as previously reported (Stewart, 2011a, 2011b). In-stream submerged, emergent and edge vegetation, small and large woody debris and snag piles were categorised into five ordinal categories (0–4) based on the proportion cover of streambed (none, 1–25%, 25–50%, 50–75% and > 75%) (Stewart, 2011b). Channel substratum was assessed by hand-judged texture and visual inspection of proportions of sand, silt, clay, gravel or rock. Stream depth and width were visually estimated at the point of fauna sampling.

Fauna sampling used standard methods described previously (Halse et al., 2007; Stewart, 2011a, 2011b). Briefly, EPT taxa were collected by kick-sampling with a 30 by 36 cm square-based net with 250-μm mesh. Debris was sorted into fractions by rinsing through 5 mm, 500 μm and 250 μm sieves and placed in white trays for live-picking by two observers for 30 min per site. Fauna samples were preserved in 70% methylated spirits.

### Species identification

All collected EPT specimens were identified to species (or lowest practical taxonomic unit) using published identification keys (Dean & Bunn, 1989; Dean & Suter, 1996; Dean et al., 2004; Dean, 1999a, 1999b; Hawking et al., 2013; Hynes & Bunn, 1984; St Clair, 2000) under a dissecting microscope (SMZ1000 with 8 × magnification, Nikon, Japan). Identifications were limited to those species known to exist in the three catchments based on previous findings or had a published distribution straddling these catchments (approximately 44 potential species) (Stewart, 2009, 2011a, 2011b; Sutcliffe, 2003).

### Data transformation and coding

Distributions of water quality variables were assessed with histograms; subsequently, EC, TN and TP were log-transformed to reduce positive skew. An overall Water Quality Index (WQI) was calculated based on the Framework for the Assessment of River and Wetland Health (FARWH) method (Storer et al., 2010) such that EC, DO, Turbidity, TN and TP were normalised on a scale of 0–1 (1 indicating best water quality, Table S1) and the final WQI was the lowest normalised score of either salinity, DO or the average of turbidity, TN or TP.

To assess degradation due to physical factors, a combined physical and fringing zone index (PFZI) was created based on the FARWH method (Storer et al., 2010). The PFZI used a Euclidian distance formula (Table S2) to incorporate the normalised scores of erosion, sedimentation, fringing vegetation and proportion vegetation cover to create one index from 0 to 1 (1 indicating least degradation) (Storer et al., 2010).

Ordinal instream habitat variables were combined to allow sufficient range to enable analysis. The instream vegetation score was the addition of submerged, emergent and edge plants, and the instream debris score was the addition of small and woody debris, leaf litter and snag piles. The substrate was coded as either gravel-containing or not since streambed organisms are often attracted to heterogenous substrates (Halse et al., 2007).

### Statistical analysis

Relationships between the EPT index (species count) and degradation gradients (hypothesis 1) were investigated using generalised linear models (GLMs) and generalised additive models (GAMs) with Poisson error distribution in R version 3.6.3. Quasi-Poisson regression was used in univariate models where overdispersion was present (if residual deviance was over twice the degrees of freedom) to adjust standard errors and *P*-values. TP had a moderate correlation with TN (Pearson’s *r* = 0.53, *P* < 0.001) and was not used in the same models to reduce collinearity. Autocorrelation of spatial data was tested in ArcGIS Pro 3.1 (ESRI, USA) using Global Moran’s *I* on deviance residuals for relevant Poisson regressions. Spatial autocorrelation was present for all Poisson GLMs: this was reduced to non-significant levels by adding easting to the models as per previously reported methods (Kefford et al., 2022), although northing could not be used to reduce autocorrelation due to high correlation with salinity gradients. Two multiple regression models were tested: one with the WQI as the main explanatory variable and one with separate water quality variables EC, DO, TN, turbidity and pH. Each initial model also contained PFZI, instream debris, instream vegetation, stream depth, stream width and gravel substrate. Bayesian information criterion (BIC) stepwise reduction was used to reduce models to those variables that showed statistical significance at 0.05 level.

Analysis of similarities (ANOSIM) tests were used to assess if there were significant differences in EPT species composition between sites with different degradation levels (hypothesis 2). The significance criteria for Global R statistic was 5%. Categories of salinity, DO, TN, WQI and PFZI score were created as per Tables S1 and S2. The ‘high salinity’ threshold of 2.1 PSU was based on previous findings where freshwater macroinvertebrate abundance and richness declined above this level (Pinder et al., 2005). Other environmental categories were created by combining high, moderate and low FARWH categories (Storer et al., 2010). Community differences used species’ abundances to create resemblance matrices using the Bray–Curtis method. Abundances were not transformed as this would emphasise rarer species which are considered less informative as bioindicators (Chessman, 2003; Lomond, 1997). Sites containing only one species not seen elsewhere, or sites without EPT taxa were removed from the dataset for composition analysis, leaving 28 taxa across 75 sites. Differences in EPT assemblage among sites were visualized by non-metric multidimensional scaling (nmMDS) and, if appropriate, the similarity percentage function (SIMPER) was used to identify individual species’ contribution to composition differences. All composition analyses were conducted using Primer Version 6 (Primer Ltd, UK).

To explore whether any individual species were tracking environmental gradients (hypothesis 3), we first identified species that appeared at more than 10% of sites and could be considered common enough to be potential indicator species in their own right. Multiple logistic regression with BIC stepwise model reduction was used to find significant relationships between these species’ presence and environmental gradients. Presence data were considered a more suitable outcome measure than abundance since some EPT taxa, particularly caddisflies, are found in low abundance (Bunn & Davies, 1992). Potential explanatory variables were the same as for Poisson regressions.

## Results

### River condition summary

Environmental variables differed by catchment (Table 1), most likely due to differing northward extents (known to be associated with salinity (Department of Water and Environmental Regulation & Department of Primary Industries and Regional Development, 2021b; Mayer et al., 2005)) and variations in land use (Stewart, 2011a, 2011b). Gravelly substratum existed in 28% of Denmark sites, 22% of Hay-Mitchell sites and 14% of Marbelup Brook sites (21% of all three catchments). With regard to degradation measures, the Denmark catchment had lower salinity, TN, TP and turbidity and higher DO than the Hay-Mitchell and Marbelup Brook catchments. Median EC values for all study catchments were above the Australia and New Zealand Environment and Conservation Council (ANZECC) trigger value for disturbed ecosystems in south-western Australia (300 μScm^−1^) while medians for DO, TN and TP were close to ANZECC trigger values (ANZECC, 2000), indicating that many sites were likely experiencing ecosystem disturbance from water quality issues. The WQI was higher for the Denmark catchment and lowest for the Hay-Mitchell: overall the WQI ranged from 0 (poorest quality) to 0.95 (high quality), indicating a range of degradation had been sampled. Similarly, the PFZI was highest for the Denmark catchment (indicating less bank modification) than Hay-Michell or Marbelup Brook, while the overall range from 0.29 to 1 indicates that high and low levels of bank modification are present in this study sample. To ensure the complete range of environmental gradients was represented, data from all three catchments were pooled for analysis.

**Table 1:** Median, interquartile range (IQR) and range of explanatory environmental variables by study catchment

	Denmark (*n* = 32)			Hay-Mitchell (*n* = 37)			Marbelup Brook (*n* = 29)			All Three catchments (*N* = 98)		
	Median	IQR	Range	Median	IQR	Range	Median	IQR	Range	Median	IQR	Range
EC (μScm^−1^)	1404	1596	543–9294	7370	7866	419–28,100	1014	707	260–2815	1521	4660	260–28100
Salinity (PSU)	0.71	0.79	0.25–5.28	4.52	4.52	0.30–17.29	0.49	0.35	0.04–1.47	0.77	2.96	0.04–17.29
pH	6.22	0.88	4.07–7.40	6.63	0.93	3.97–8.01	6.29	0.83	4.36–6.99	6.41	0.91	3.97–8.01
Dissolved oxygen (ppm)	8.33	1.5	5.10–10.87	8.79	1.94	5.20–12.00	8.80	2.25	3.80–11.90	8.80	1.68	3.80–12.00
Dissolved oxygen (%)	79.4	11.2	48.0–95.8	81.9	16.3	49.4–106.1	80.0	15.2	35.7–103.4	81.2	12.9	0–106.1
Turbidity (NTU)	7.75	6.32	0–54.00	9.70	19.50	0–168.00	15.00	29.80	0–254.90	9.65	16.30	0–254.90
TN (μgL^−1^)	765	543	234–4966	1060	935	118–4616	1002	1693	186–9620	844	979	118–9620
TP (μgL^−1^)	17.0	15.5	0–227.0	10.0	19.0	0–1644.0	65.5	246.0	0–1204.0	17.5	38.9	0–1644.0
Water quality index	0.83	0.15	0.50–0.95	0.50	0.25	0–0.95	0.75	0.25	0.40–0.95	0.75	0.35	0–0.95
Physical and Fringing Zone Index	0.80	0.21	0.32–1.00	0.69	0.28	0.35–1.00	0.55	0.29	0.29–1.00	0.70	0.32	0.29–1.00
Debris score	6	3.25	1.0–12	5	4	2–14	2	2	0–6	4	3.75	0–14
Plant score	4	2	1–9	4	2	1–7	4	3	0–8	4	2	0–9
Channel depth (cm)	22.5	30	5.0–150	30	45	5.0–100	30	30	10–80	30	40	5.0–150
Channel width (m)	1.6	1.6	0.4–20	2.0	2.0	0.40–15	1.5	1.5	0.60–10	1.88	2.0	0.4–20

### EPT fauna summary

Sampling identified ten EPT families with 35 distinguishable taxa. Twenty-six taxa were identified at the species level, five taxa were identified at the genus level and four caddisflies were not able to be identified but were visually distinct from each other and all other specimens. Overall, 22% of sites had no EPT fauna (Table 2). Of the 26 identifiable species, 19 (73%) are endemic to south-western Australia, two are endemic to Western Australia and five have Australia-wide distributions (Dean, 1999a, 1999b; Stewart, 2009; Sutcliffe, 2003; Suter, 1999).

**Table 2:** Summary of EPT species richness across study catchments

Catchment	Taxa group	Median/site	Maximum/site	*N* (%) sites with no EPT	Total species in the catchment
Denmark (*n* = 32)	All EPT	2	6	6 (19)	23
	Ephemeroptera	0	3	23 (72)	5
	Plectoptera	0	2	17 (53)	3
	Trichoptera	1	3	11(34)	15
Hay-Mitchell (*n* = 37)	All EPT	1	4	13 (35)	21
	Ephemeroptera	0	2	32 (86)	3
	Plectoptera	0	2	22 (59)	3
	Trichoptera	1	4	18 (49)	15
Marbelup Brook (*n* = 29)	All EPT	4	8	3 (10)	24
	Ephemeroptera	0	3	11 (38)	5
	Plectoptera	0	2	16 (55)	3
	Trichoptera	2	6	5 (17)	16
Total study area (*N* = 98)	All EPT	2	8	22 (22)	35
	Ephemeroptera	0	3	66 (67)	6
	Plectoptera	0	2	55 (56)	3
	Trichoptera	1	6	34 (35)	26

### Relationships between EPT species richness and environmental gradients

**Fig. 2:**
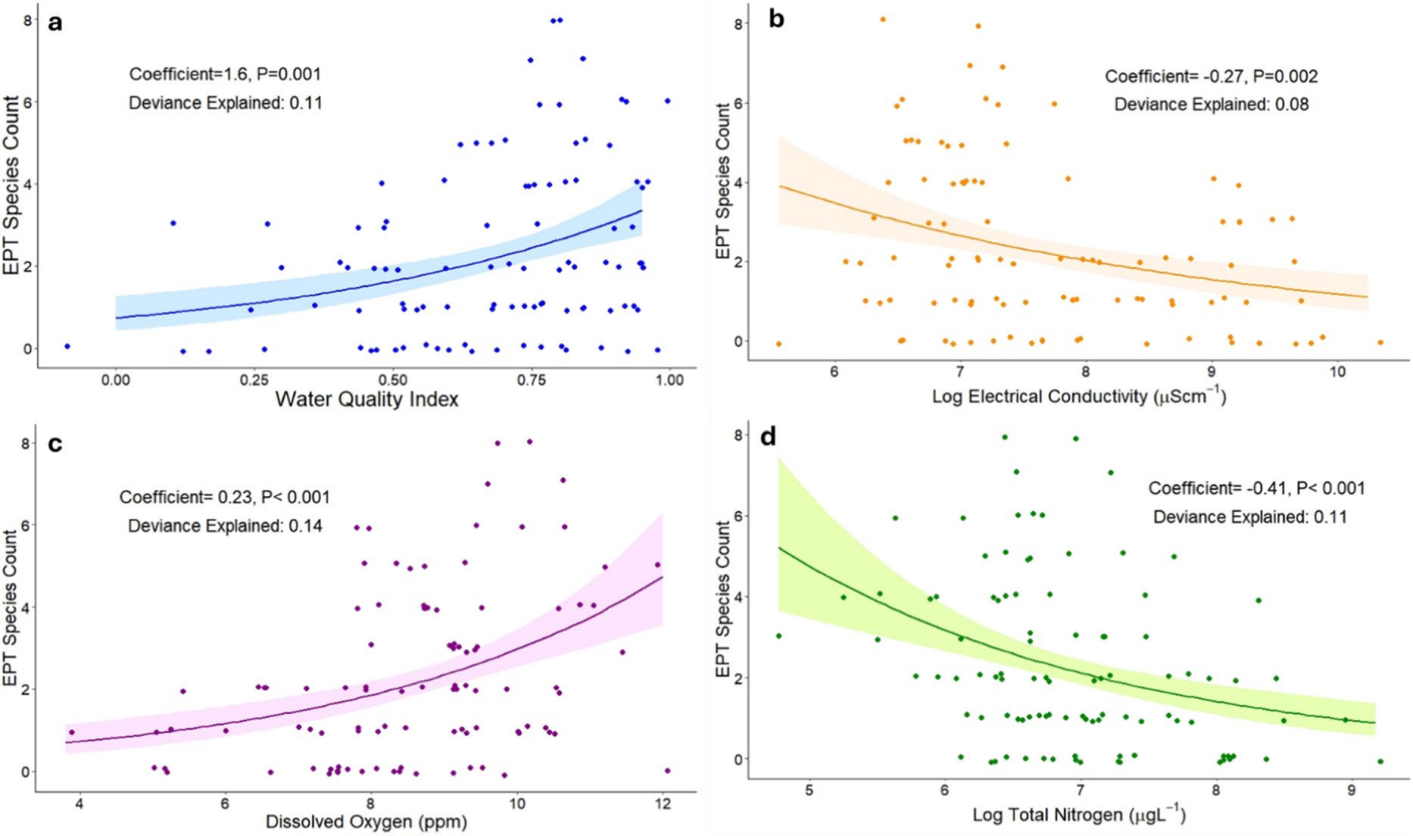
Scatterplots showing significant univariate relationships between EPT species count and Water Quality Index (**a**) log conductivity (**b**), dissolved oxygen (**c**) and log total nitrogen (**d**). Plots jittered to show all sites, *N* = 98, trend lines and 95% confidence intervals based on generalised linear models with quasi-Poisson error distribution

The results of univariate regression showed significant but weak positive relationships for WQI and DO and weak negative relationships for log EC and log TN (Fig. 2A–D). Precision for all these variables tended to decrease with higher water quality and each only explained 5% to 14% of the variance (Fig. 2A–D). Turbidity, pH, PFZI, instream debris, instream plants, width and gravel substrate did not show any significant univariate relationship with the EPT index in GLM or GAM models. Channel depth had a significant positive relationship with the EPT index on both GLM and GAM (GAM trend levelling off after 50 cm depth).

Two multiple regression models were produced by BIC reduction. In Model 1, the WQI still showed a weak significant positive relationship with EPT species richness: the reduced model also retained stream depth and Easting (Table 2). Model 2 retained log EC and log TN (negative relationships) and DO (positive relationships) as well as depth and Easting (Table 3). Log turbidity, pH, PFZI score, debris score, instream plant score, stream width and substrate type were not significantly related to EPT species richness and were not retained in the final models.

**Table 3:** Multiple regression models of EPT species richness by water quality and habitat variables

	Variable	Coefficient^a^	s.e.^a^	*z*-value	*P*-value	Deviance explained
Model 1	WQI	2.16	0.38	5.66	< 0.0001	0.32
	Depth (cm)	8.1 × 10^−3^	2.2 × 10^−3^	3.67	0.0002	
	Easting	2.2 × 10^−5^	4.2 × 10^−6^	5.41	< 0.0001	
Model 2	Log EC (μScm^−1^)	− 0.25	0.07	− 3.64	0.0003	0.41
	DO (ppm)	0.14	0.05	2.89	0.004	
	Log TN (μgL^−1^)	− 0.43	0.10	− 4.34	< 0.0001	
	Depth (cm)	6.7 × 10^−3^	2.3 × 10^−3^	2.89	0.004	
	Easting (m)	1.9 × 10^−5^	4.4 × 10^−6^	4.46	< 0.0001	

^a^Estimates and standard errors (s.e.) derived from multiple generalised linear models with Poisson distribution (log function). Initial models included water quality index (Model 1) or individual water quality measures (log EC, DO, log turbidity, log total nitrogen, pH, Model 2), physical and fringing zone index, instream debris, instream plants, channel depth and width, substrate and easting. Final models obtained by Bayesian information criterion stepwise reduction

### EPT community composition

**Fig. 3:**
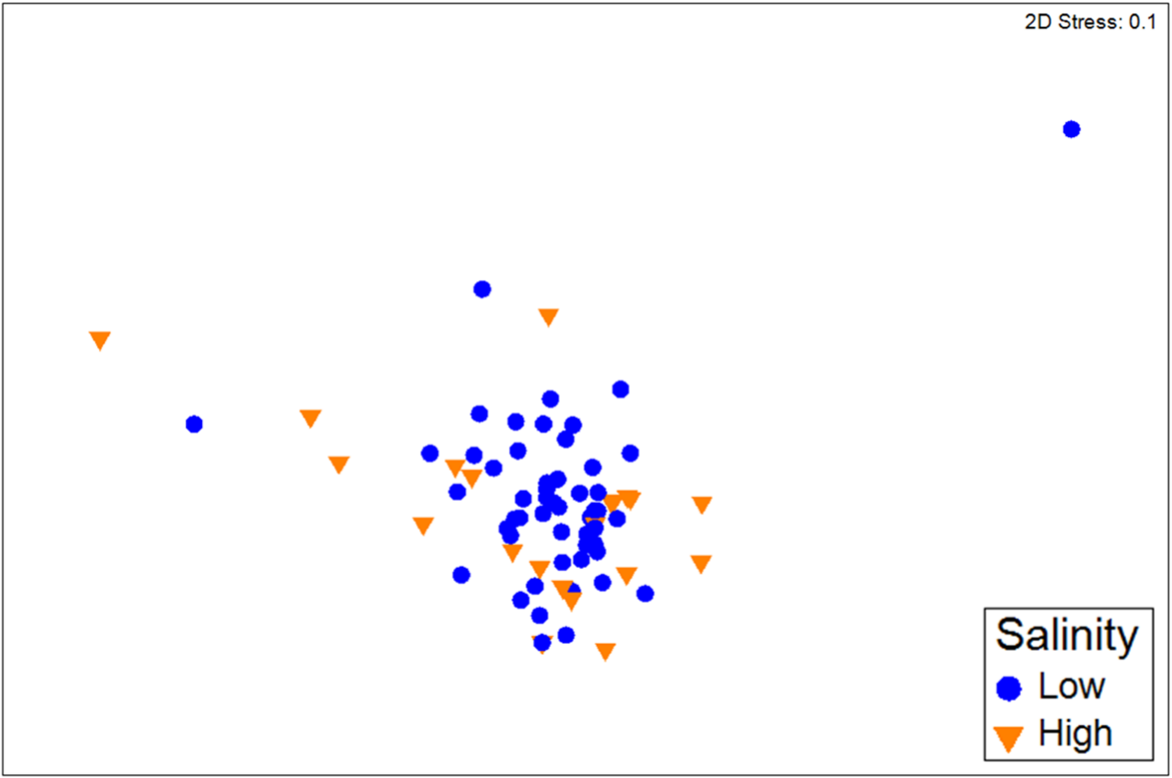
Non-metric multidimensional scaling ordination of EPT composition data. The resemblance matrix used non-transformed Bray Curtis similarity, *N* = 75 sites. Markers are coloured by salinity above or below 2.1 practical salinity units

EPT community structure did not readily distinguish between river quality parameters, as demonstrated by ANOSIM tests with low Global R scores (Table 4). Salinity had the highest Global R of 0.14 and was the only measure where further analysis was deemed appropriate. Visual inspection of non-metric MDS ordination showed that there was considerable overlap between sites labelled by high and low salinity (Fig. 3). SIMPER analysis showed that the highest contributor to composition differences by salinity level was *Newmanoperla exigua*, and along with *Notalina* sp. AV15 and *Tasmanocoenis tillyardi*, this species was more abundant at high salinity sites (Table 5). Three other species showed lower abundance at high salinity, with the remaining 30% contribution to dissimilarity being made up of ten species that contributed less than 5% each.

**Table 4:** Results of ANOSIM tests for EPT species composition differences by environmental category (*N* = 75 catchments)

Environmental group	Global R	Sig. of R
Water Quality Index	0.07	2.5%
Physical and Fringing Zone Index	0.05	4.6%
Salinity	0.14	0.5%
Dissolved oxygen	0.09	1.0%
Total nitrogen	0.04	6.7%
Total phosphorous	-0.03	78.2%

**Table 5:** Results of SIMPER analysis of individual species contribution to differences in community structure by salinity level

Species	Average abundance (*N* = 75 sites)		% contribution	Cumulative contribution
	Salinity < 2.1 PSU	Salinity ≥ 2.1 PSU		
*Newmanoperla exigua*	4.8	11.9	27.8	27.7
*Nyungara bunni*	6.6	0	8.7	36.5
*Notalina sp. AV15*	0.5	1.7	8.6	45.1
*Leptoperla australica*	2.1	2.3	7.5	52.5
*Lectrides parilis*	1.4	0.6	5.9	58.4
*Tasmanocoenus tillyardi*	0.5	4.5	5.7	64.1
*Hudsonema aptus*	3.1	0.1	5.2	69.3

### Individual species’ response to environmental gradients

Only seven species were present in at least 10% of sites: *N. bunni* (at 17% of sites), *T. tillyardi* (10%), *Bibulmena kadjina* (10%), *N. exigua* (40%), *H. aptus* (21%)*, L. parilis* (17%) and *Notalina* sp.AV15 (17%).

The results of multiple logistic regression models reduced by BIC (Table 6) showed that response to degradation differed among species. Both *N. bunni* and *H. aptus* were more likely to occur at sites with low salinity and high dissolved oxygen, with *N. bunni* additionally more likely to occur at sites with low TN. Both these species’ models explained approximately half of the variance. *N. exigua* and *L. parilis* occurred more frequently at sites with high dissolved oxygen and *B. kadjina* at low salinity; however, the models only explain up to a quarter of the deviance. Despite the tendency to be more abundant with higher salinity (Table 5), the overall presence of *N. exigua* and *Notalina* sp. AV15 was not significantly associated with salinity (Table 6). *N. bunni* and *B. kadjina* were completely absent from sites with over 3000 μScm^−1^ or 1400 μScm^−1^ conductivity respectively.

**Table 6:** Multiple logistic regression models for the presence/absence of seven species by significant environmental variables

Species	Final model variables^a^	Coefficient	s.e	Odds ratio	*z*-value	*P*-value	Deviance explained
*Nyungara bunni*	Log EC (μScm^−1^)	− 1.70	0.63	0.2	− 2.70	0.007	0.50
	DO (ppm)	0.76	0.34	2.1	2.21	0.027	
	Log TN (μgL^−1^)	− 2.18	0.79	0.1	− 2.77	0.006	
	Debris	− 0.64	0.23	0.5	− 2.78	0.005	
	Depth (cm)	0.05	0.02	1.1	2.45	0.014	
	Gravel Substrate	2.90	1.19	18.2	2.44	0.015	
*Bibulmena kadjina*	Log EC (μScm^−1^)	− 1.79	0.63	0.2	− 2.86	0.004	0.25
	pH	1.97	0.79	7.2	2.50	0.013	
*Newmanoperla exigua*	DO (ppm)	0.40	0.15	1.5	2.62	0.009	0.06
*Hudsonema aptus*	Log EC (μScm^−1^)	− 0.81	0.37	0.4	− 2.16	0.031	0.48
	DO (ppm)	0.93	0.30	2.5	3.15	0.002	
	Instream vegetation	0.43	0.22	1.5	1.98	0.047	
	Easting (m)	1.3 × 10^−4^	3.5 × 10^−5^	1.0	3.87	< 0.001	
*Lectrides parilis*	DO(ppm)	0.68	0.24	2.0	2.84	0.004	0.25
	Easting	6.9 × 10^−5^	2.3 × 10^−5^	1.0	3.00	0.003	
*Tasmanocoenis tillyardi*	Instream vegetation	− 0.57	0.22	0.6	− 2.5	0.012	0.12
*Notalina* sp. AV15	Easting (m)	− 7.5 × 10^−5^	2.3 × 10^−5^	1.0	− 3.30	0.001	0.17

^a^Coefficients, odds ratios and standard errors (s.e.) estimated using generalised linear regression (binomial error distribution, logit function) reduced by Bayesian information criterion. Initial models included all listed variables as well as physical and fringing zone index, log turbidity and stream width

## Discussion

The EPT index is a globally-known, rapid bioassessment that has successfully tracked river pollution and degradation in eastern Australia (Growns et al., 1997; Kefford et al., 2022; Metzeling et al., 2006; Wright & Burgin, 2009), but its capacity to track river degradation in south-western Australia is unknown. Our first hypothesis that the EPT species count would track degradation gradients was weakly supported: we found that EPT species richness was higher with higher overall water quality, higher oxygen, lower salinity and lower total nitrogen, but this result was tempered with weak effect sizes and a high frequency of low or zero EPT counts across the range of water quality parameters. Additionally, EPT species richness did not seem to respond to measures of physical and riparian vegetation or turbidity. Similarly, the second hypothesis that community composition would differ between high- and low-quality sites was only weakly supported. Salinity had the highest effect size, but EPT communities were still too poorly differentiated to be useful for bioassessment. Our third hypothesis that individual species may track gradients was also supported in that most species investigated were more likely to appear at high oxygen sites and less likely to appear at high salinity sites. However, few species were common enough to be useful bioindicators, and some species responded unexpectedly to gradients of water quality.

The endemic mayfly *N. bunni* tracked salinity, oxygen and nitrogen in expected directions but had a very restricted distribution which could be due to degradation levels, natural habitat preferences or biogeography. This species’ sensitivity to poor water quality is in agreement with previous studies where this species preferred fast-flowing streams and avoided low oxygen conditions (Storey et al., 1991). A study on the Richmond River in New South Wales found Leptophlebiidae mayflies (the same family containing *N. bunni* and *B. kadjina*) were largely absent from degraded sites (Cox et al., 2019). Caenid mayflies have less consistent sensitivity, with the genus *Tasmanocoenis* being absent from high salinity sites in the Western Australian wheatbelt (Pinder et al., 2005), but the same genus showing apparent tolerance to sediment and mining waste in south-western Australia (Bunn et al., 1986). Caddisflies whose presence tracked environmental gradients in the current study (*L. parilis* and *H. aptus*) are common in south-western Australia (Stewart, 2009) but generally found in low abundance (Bunn & Davies, 1992). These species’ absence from the Eastern South Coast Bioregion supports the current finding that they are intolerant of high salinity (Stewart, 2009). These two caddisflies may be worthy of further investigation as bioindicators, but only in rivers of the Western South Coast bioregion.

Of interest was that one of our species appeared to be markedly tolerant of poor water quality. The widespread stonefly *N. exigua* was present at a range of salinities and was also a key driver of composition differences by salinity (where it was more abundant at high salinity). This species has previously shown insensitivity to high salinity (Storey et al., 1990) as well as low-flow conditions caused by damming (Storey et al., 1991) and turbidity caused by forestry (Growns & Davis, 1994). While stoneflies are considered sensitive to water quality in other regions (Chessman, 2003; Lomond, 1997), in south-western Australia, this common species may be more likely to weaken the overall strength of the EPT index due to its tolerance to salinity or other degradation indicators. Our study indicates that sensitivity grades allocated to eastern states EPT families may not be appropriate for south-western EPT taxa. For example, the SIGNAL2 (Stream Invertebrate Grade Number Average Level) biotic index allocates sensitivity grades to macroinvertebrate families from 1 (insensitive) to 10 (most sensitive), with high-quality sites having a high average grade (Chessman, 2003). SIGNAL2 gives the stonefly family Gripopterygidae a grade of 8 (Chessman, 2003); however, in the current study, the common Gripopterygid stonefly *N. exigua* seemed insensitive to most water quality measures.

Although many studies in eastern Australia have successfully used the EPT index to track degradation (Chessman et al., 2006; Growns et al., 1997; Metzeling et al., 2006; Wright & Burgin, 2009), some Australian studies have indicated problems with the index in certain circumstances and locations. In Victoria, where the EPT fauna is so rich, they only need to be identified at the family level; EPT were found to be too sparse in the Murray Plains and highly modified lowland sites to be useful as a biotic index (Metzeling et al., 2006). A study in NSW also found that while the EPT index was effective in elevated locations, it could not track degradation in lowland, arid-country rivers with low flow (Chessman et al., 2006). The EPT index was developed in the Northern Hemisphere where fast-flowing, perennial, gravelly streams are common — previous authors have suggested that its use as a bioindicator cannot be assumed in Australia where aridity and intermittent stream flow are commonplace (Bunn & Davies, 1990; Chessman et al., 2006).

There are several possible reasons why the EPT index failed to show strong links to gradients of environmental damage and water quality in this study. Firstly, the EPT fauna in south-western Australia is depauperate (Bunn & Davies, 1990), with only around 44 species predicted to exist in the study catchments (Stewart, 2009, 2011a, 2011b; Sutcliffe, 2003). Sampling will never capture the maximum theoretical species available, especially if many species are rare. In this study, only seven species were found at more than 10% of sites, making reliability and repeatability of results difficult. Low species counts result in higher error and reduce the range of values with which to track gradients (Metzeling et al., 2006). Low counts could be mitigated by aggregation of sites, but then the association with local degradation could be lost. Secondly, the insensitivity to or apparent preference for high salinity sites from some species will dilute the effectiveness of the EPT species count. Thirdly, if individual species become very rare in a region, then their utility to distinguish between sites with moderate versus severe degradation is limited (Bunn et al., 1986; Metzeling et al., 2006). A biotic index needs to be comprised of species that are known to be naturally common in a region of interest; otherwise, there may be too many sites where no members of the index occur and results are uninterpretable. Lastly, sampling error and environmental conditions not related to degradation also reduce bioindicator effectiveness (Halse et al., 2007; van Looij, 2009). For example, sampling at some sites was hindered by high loads of fine debris (especially in streams passing through paperbark melaleuca thickets) or discoloured water, making it difficult to ensure all EPT fauna had been found in 30 min. Heavy rain was experienced in early June 2023, which may flush through macroinvertebrate populations for a period and temporarily lower species richness (Halse et al., 2007; Kay et al., 2001; van Looij, 2009). EPT abundance and body size may also be higher in late spring (September–October) (van Looij, 2009), while the data for this study were obtained mainly in early to mid-winter. It is possible that higher species richness would be seen in spring due to both increased abundance and the increased ease of spotting larger individuals.

Other limitations of this study include the single time period of sampling: since natural seasonal variation will occur as stream flow and emergence conditions change (Bunn & Davies, 1990; Bunn et al., 1986; Lenat & Barbour, 1994), it would be useful to replicate this work over multiple seasons to assess natural temporal variability in south-western rivers. For logistical reasons, sites were limited to those that were wadable and accessible by road and may not fully represent all possible stream conditions in the study site. Dissolved oxygen was taken at one time point, but this measure naturally decreases at night: daytime sampling is likely to give a best-case scenario for oxygen levels (Storer et al., 2010). Flow velocity was also not systematically collected in this study, and while this is likely to correlate with DO, its inclusion in future studies may help delineate species’ habitat preferences.

This study identifies several avenues for future research. It is possible that a viable modification of the EPT index could still provide useful data, but south-western species would have to be researched and selected based on reliable estimates of their sensitivity to key degradation measures. Indicator species need to avoid having highly restricted distributions or be insensitive to key measures of water quality such as has arisen in the current study. This study has also shown that different species could be suitable for different research questions, as species sensitive to salinity may be tolerant to excess nutrients and vice versa. With family richness being low, any designated grades similar to SIGNAL2 should be calculated for local fauna and at genus or species-level resolution in south-western Australia.

Investigation of the distributional range (especially inland extent) of common species in relatively undisturbed streams may make it easier to separate biogeographical and habitat-related distribution from changes due to human-induced degradation. Additionally, there are potentially more EPT species in south-western Australia that are only known by voucher specimens or are not represented in taxonomic keys (Cartwright et al., 2023; Dean, 1999a) — further taxonomic work and nymph descriptions could help clarify the true species richness and aid identification (Braby & Williams, 2016).

## Conclusions and recommendations

With rivers and streams in south-western Australia coming under increasing threat of habitat degradation, timely and effective monitoring is critical to ensure changes are promptly identified and appropriate action is taken. The key finding of this South Coast case study is that both EPT species richness and composition were only very weakly associated with habitat quality parameters, and as such, the use of the EPT index for tracking river conditions in south-western Australian systems could lead to misleading indications of river habitat quality. Bioassessment needs robust measures due to the inherent variability in river conditions, so in this study, we have interpreted results conservatively. It is possible that the EPT index would need to be bolstered by other sensitive macroinvertebrates that are common in the region. The results of this study lead to three major recommendations for further research. Firstly, it might be useful to assign endemic EPT and other macroinvertebrate species a meaningful sensitivity grade (similar to those in SIGNAL2, based on targeted research for regional conditions (Chessman, 2003)). Secondly, while distributions of stoneflies and caddisflies have been well reported (Sutcliffe, 2003), the natural distributions of endemic mayflies need clarification so that local biogeography can be accounted for when interpreting findings. Thirdly, the variability of species richness by season or weather conditions could be established at sites known to house EPT, so this could be accounted for when measuring degradation. For all these recommendations, the current study may provide useful pilot data to establish the likely presence of key species, reference sites and expected variability between sites.

### Supplementary Information

Below is the link to the electronic supplementary material.

Supplementary file1 (DOCX 883 KB)

## Data Availability

Data used in this study available from the corresponding author Barbara Stewart on reasonable request.

## Declarations

Ethical approval.

All authors have read, understood and have complied as applicable with the statement on “Ethical responsibilities of Authors”. Animal Ethics approval was not required for this research. Permission to take fauna and enter reserves was granted by the Department of Biodiversity, Conservation and Attractions, Licence number FO25000471.
