# Supplementary material for: Can a naturally depauperate Ephemeroptera, Plectoptera and Trichoptera (EPT) fauna track river degradation in south-western Australia?
Source: Environ Monit Assess. 2024 Jun 3;196(7):592. doi: 10.1007/s10661-024-12734-8 (PMC11147854; doi:10.1007/s10661-024-12734-8)
Supplement: Supplementary file 1 — Supplementary file1 (DOCX 883 KB) [file 10661_2024_12734_MOESM1_ESM.docx]

**Supporting Information**

**Can a naturally depauperate Ephemeroptera, Plectoptera and Trichoptera (EPT) fauna track river degradation in south-western Australia?**

**Kathryn R. Greenop^1, 2^, Paul G. Close^2, 3^ and Barbara A. Stewart^2, 3^**

1: School of Biological Sciences, University of Western Australia, Perth, Western Australia, 6009, Australia

2: Centre for Natural Resource Management, University of Western Australia, Albany, Western Australia, 6330, Australia

3: School of Agricultural and Environmental Science, University of Western Australia, Albany, Western Australia 6330, Australia

Email of corresponding author (Barbara Stewart): Barbara.cook@uwa.edu.au

List

Table S1: Water chemistry normalization, categories and Water Quality Index calculation

Table S2: Physical and vegetation normalized scores and Physical and Fringing Zone Index calculation

Fig. S1: Presence or absence of five key mayfly, stonefly and caddisfly species for 98 study sites in three catchments on the South Coast of Western Australia.

**Table S1** Water chemistry normalization, categories and Water Quality Index calculation

| **Variable** | **Normalized Score** | **Categorized group** |
| --- | --- | --- |
| Conductivity μScm^-1^ |  |  |
| <500 | 1.0 |  |
| 500-1000 | 1.0 |  |
| 1000-1500 | 0.9 |  |
| 1500-3000 | 0.8 |  |
| 3000-7000 | 0.5 |  |
| 7000-14500 | 0.2 |  |
| >14500 | 0 |  |
| <2.1 PSU |  | Low Salinity |
| ≥2.1 PSU |  | High Salinity |
| Dissolved oxygen parts per million |  |  |
| >6 | 1 |  |
| 5-6 | 0.8 |  |
| 4-5 | 0.6 |  |
| 3-4 | 0.4 |  |
| 2-3 | 0.2 |  |
| <2 | 0 |  |
| <8 |  | Low Oxygen |
| 8-8.9 |  | Moderate Oxygen |
| >9 |  | High Oxygen |
| Total nitrogen μgL^-1^ |  |  |
| <750 | 1 |  |
| 750-1200 | 0.8 |  |
| 1200-2000 | 0.6 |  |
| >2000 | 0.4 |  |
| <750 |  | Low Nitrogen |
| 750-1500 |  | Moderate Nitrogen |
| >1500 |  | High Nitrogen |
| Total phosphorous μgL^-1^ |  |  |
| <20 | 1 |  |
| 20-80 | 0.8 |  |
| 80-200 | 0.6 |  |
| >200 | 0.4 |  |
| <20 |  | Low Phosphorous |
| 20-50 |  | Moderate Phosphorous |
| >50 |  | High Phosphorous |
| Turbidity NTU |  |  |
| <5 | 1 |  |
| 5-10 | 0.8 |  |
| 10-25 | 0.6 |  |
| >25 | 0.4 |  |
| Water Quality Index^a^ | 0.80-1.0 | High quality |
|  | 0.60-0.79 | Moderate quality |
|  | 0-0.59 | Poor quality |

^a^Final Water Quality Index is LOWEST of either DO, EC, or average of (Turbidity, TN, TP) normalized scores, based on Framework for Assessment of River Health (Storer et al., 2010).

**Table S2** Physical and vegetation normalized scores and Physical and Fringing Zone Index calculation

| **Physical variable** | **Normalized score** | **Categorized group** |
| --- | --- | --- |
| Bank erosion |  |  |
| 0-5% | 1 |  |
| >5-20% | 0.75 |  |
| >20-50% | 0.25 |  |
| >50% | 0 |  |
|  |  |  |
| Sedimentation |  |  |
| 0-5% | 1.0 |  |
| 5-20% | 0.8 |  |
| 20-50% | 0.6 |  |
| >50% | 0.2 |  |
|  |  |  |
| Width Fringing Vegetation |  |  |
| >100m | 1.0 |  |
| 20-100m | 0.8 |  |
| 5-20m | 0.6 |  |
| <5m band | 0.4 |  |
| Absent | 0 |  |
|  |  |  |
| % Exotics or weeds |  |  |
| 0-5% | 1.0 |  |
| >5-20% | 0.8 |  |
| >20-50% | 0.6 |  |
| >50-75% | 0.2 |  |
| >75% | 0.1 |  |
|  |  |  |
| Bank stabilization (proportion tree/shrub cover) |  |  |
| >75% | 1.0 |  |
| 50-75% | 0.8 |  |
| 25-50% | 0.6 |  |
| 1-25% | 0.4 |  |
| 0% | 0 |  |
|  |  |  |
| PFZI^a^ | ≥0.8 | Largely unmodified |
|  | 0.5-0.79 | Moderate-slight modification |
|  | <0.5 | Severe-substantial modification |

^a^Physical and Fringing Zone Index (PFZI) = 1-(√(1-erosion)^2^ +√(1-sedimentation)^2^ +√(1-width fringing vegetation)^2^ +√(1-bank stabilization)^2^ )/√5 (calculation based on Framework for Assessment of River Health (Storer et al., 2010))


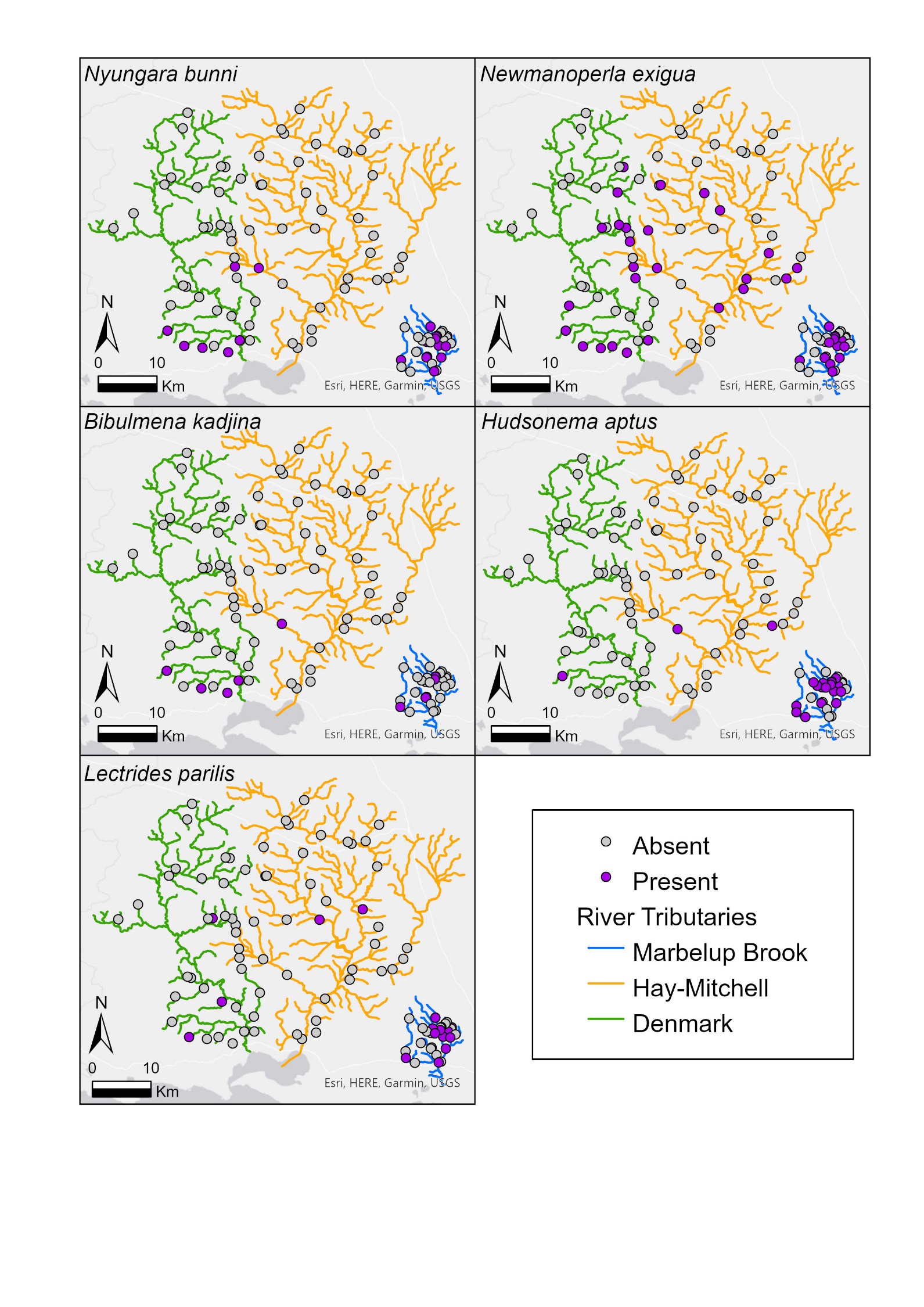


**Fig. S1** Presence or absence of five key mayfly, stonefly and caddisfly species for 98 study sites in three catchments on the South Coast of Western Australia.
